# Free Space Measurement Device Independent Quantum Key Distribution with Modulating Retro-Reflectors under Correlated Turbulent Channel

**DOI:** 10.3390/e23101299

**Published:** 2021-10-01

**Authors:** Xingyu Wang, Wei Liu, Tianyi Wu, Chang Guo, Yijun Zhang, Shanghong Zhao, Chen Dong

**Affiliations:** 1 Information and Communication College, National University of Defense Technology, Xi’an 710006, China; wang_kgd@foxmail.com (X.W.); liuwei0927@126.com (W.L.); wutianyi13@nudt.edu.cn (T.W.); gxcy185@163.com (C.G.); gfkd_zyj@163.com (Y.Z.); 2 School of Information and Navigation, Air Force Engineering University, Xi’an 710077, China; zhaoshangh@aliyun.com; 3 Graduate Institute, Rocket Force University of Engineering, Xi’an 710025, China

**Keywords:** MDI-QKD, modulating retro-reflector, double-pass channel, atmosphere turbulence

## Abstract

Modulating retro-reflector (MRR), originally introduced to support laser communication, relieves most of the weight, power, and pointing requirements to the ground station. In this paper, a plug-and-play measurement device independent quantum key distribution (MDI-QKD) scheme with MRR is proposed not only to eliminate detector side channels and allow an untrusted satellite relay between two users, but also to simplify the requirements set-ups in practical flexible moving scenarios. The plug-and-play architecture compensates for the polarization drift during the transmission to provide superior performance in implementing the MDI-QKD on a free-space channel, and the MRR device is adopted to relax the requirements on both communication terminals. A double-pass correlated turbulent channel model is presented to investigate the complex and unstable channel characteristics caused by the atmospheric turbulence. Furthermore, the security of the modified MDI-QKD scheme is analyzed under some classical attacks and the simulation results indicate the feasibility under the situation that the system performance deteriorates with the increase of fading correlation coefficient and the turbulence intensity, which provides a meaningful step towards an MDI-QKD based on the moving platforms to join a dynamic quantum network with untrusted relays.

## 1. Introduction

Measurement device independent quantum key distribution (MDI-QKD) [1], which is immune to all attacks against the detection system and allows a QKD network with untrusted relays, has been a promising area to guarantee the information security of communications [2,3,4]. Recently, the fiber-based implementations rapidly developed towards longer distance [5,6,7], and higher key rates [8,9]. However, the implementation of MDI-QKD requires the indistinguishability of the spectral, polarization and temporal modes of the photons from Alice and Bob, which is much more difficult to manipulate under a free space channel than a fiber-based channel because the free-space optical channels dramatically fluctuate, caused by the atmospheric turbulence [10].

An achievement of the free-space MDI-QKD experiment was made in [11], which paves a significant step towards the satellite-based MDI-QKD [12] or other QKD applications [13]. However, the links in these experiments are relatively fixed while in the satellite-to-ground scenario they are dynamic and flexible [14]. In addition, the observation of high-visibility Hong-Ou-Mandel (HOM) interference requires the indistinguishability of optical pulses that are generated by two independent photon sources and transmitted through two independent free-space channels, which is a big challenge to share the time and frequency via free-space links.

Recently, inspired by the multidirectional links using the modulating retro-reflector (MRR) for high rate free-space optical communication [15], a laboratory-based free-space QKD with the MRR setting was presented in [16], which can ease the pointing requirements and maintain the narrow beam divergence necessary for long-range communication links. Meanwhile, the fading channel of double-pass MRR free-space optical (FSO) systems under weak turbulence conditions is investigated in [17], which can be modeled by the distribution of the weighted product of two correlated Log-normal random variables. These research results lay a foundation for extending the MRR device to the MDI-QKD scheme, which can be a desirable yet highly challenging application towards future free-space MDI-QKD experiments.

In this paper, a plug-and-play MDI-QKD scheme with modulating retro-reflectors is proposed not only to inherit the merit of the structure, where the plug-and-play architecture compensates for the polarization drift during the transmission to provide superior performance in implementing the MDI-QKD on free space channel, but also to bring advantages of the MRR device, which is adopted in classical free space communication system to relax the requirements on both communication terminals. Then, considering that the fading of the two passes is correlated in the double-pass scheme, a correlated turbulent channel model for the double-pass MRR QKD link is used to investigate the turbulence effect on the key generation rate and the QBER. Furthermore, the security of the modified MDI-QKD scheme is analyzed under some classical attacks. The simulation results indicate the feasibility of the modified MDI-QKD scheme under the situation that the system performance deteriorates with the increase of the fading correlation coefficient and the turbulence intensity, which is a meaningful step to make our modified MDI-QKD with MRR suitable for mobile scenarios with flexible deployment.

The organization of the article is as follows. In Section 2, we present the concept of the free-space MDI-QKD with modulating retro-reflectors and introduce the different features in the process of the “Preparation” and “Measurement”. Then, in Section 3, the channel of the double-pass MRR-MDI-QKD link under turbulence is modeled, and the key rate of MRR-MDI-QKD is estimated by combining the correlated Log-normal distribution model and the decoy-state QKD method. In Section 4, we simulated the performance of the involved MRR-MDI-QKD. We concluded this paper in Section 5. The article is ended in with a security analysis of the MRR-MDI-QKD.

## 2. The Concept of the Free-Space MDI-QKD with Modulating Retro-Reflectors

**Figure 1:**
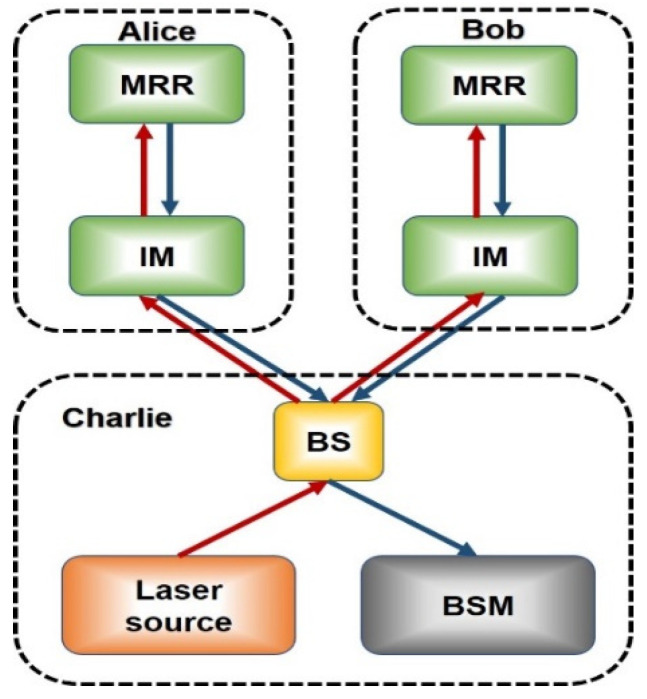
Schematic Diagram of the MDI-QKD with MRR. IM: Intensity Modulator; MRR: Modulating Retro-Reflectors; BSM: Bell State Measurement; BS: Beam Splitters.

The schematic diagram of our MRR-MDI-QKD model is shown in Figure 1. Different from the traditional MDI-QKD scheme using two laser sources from Alice and Bob respectively, the plug-and-play MDI-QKD [18] scheme with the MRR only adopts a single laser source held by Charlie, which naturally solves the problem of spectrum matching owing to the independent photon preparation process. The preparation process is achieved by using MRR and polarization beam splitters (PBS) acting on the input pulses at Alice and Bob independently and reflect the polarization-encoded photon back to Charlie to perform the measurement. The Bell state measurement (BSM) procedure is the same as the traditional MDI-QKD scheme.

**Figure 2:**
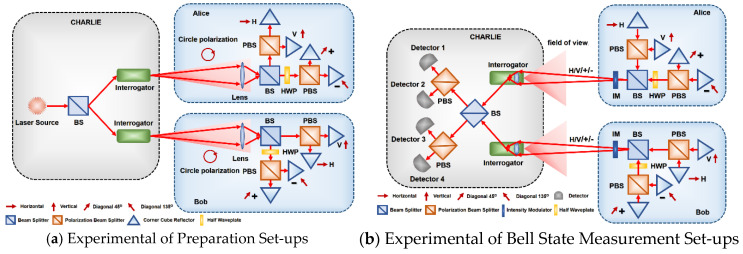
The schematic diagram of the MDI-QKD with MRR.

Similar to the original MDI-QKD protocol, Charlie sends a laser pulse through BS to split two beams into the input of interrogator. As shown in Figure 2a, the circle polarization beam sends to Alice and Bob to perform the encode process respectively.

Take the experimental apparatus of the upper part as an example; the circularly polarized beam enters Alice’s set-up diagram and is divided into two paths by a beam splitter. In the path for the H-V basis where  |H〉  and  |V〉  refer to the horizontal and vertical polarization directions, respectively, the circularly polarized beam is split into horizontal and vertical components by a polarizing beam-splitter. Alice changes the strength of transmission state of MRR to encode the H-V basis on reflected light. The process is driven by a random data signal consisting of a binary one and zero, where the modulator is driven to its high transmission state when the data consists of a binary one and the modulator is driven to its low transmission state when the data consists of a binary zero. In the path for the diagonal basis, where  |+〉  and  |−〉  |−〉  denote 45-degree and 135-degree diagonal polarization directions, Alice only needs to use half-wave plates (HWP) to apply polarization rotations to implement the process. Thus, Alice and Bob use the MRR to randomly choose a basis from  {|H〉,|V〉}  or  {|+〉,|−〉}  and reflect back the encoded photon to Charlie’s interrogator to proceed to the measurement step. In addition, an intensity modulator (IM) driven by a quantum random number generator is used to generate decoy states of the pulses to defend against a photon number splitting attack. (See Appendix A for a full description of the security analysis.)

In Figure 2b, the reflected qubits are received to the interrogator, where the BSM in our MDI-QKD implementation protocol can be conveniently performed in the polarization space the same as for the classical MDI-QKD. A successful BSM result corresponds to the observation of precisely two detectors being triggered. From all possible events of separated clicks, the two Bell basis $\;|\psi^{+}\rangle$ and $|\psi^{-}\rangle$ can be deterministically identified:

(1) $$\left(H,H\right)or\left(V,V\right)\to |\psi^{+}\rangle ,\left(H,V\right)or\left(V,H\right)\to |\psi^{-}\rangle$$

## 3. The Framework for the Key Rate Estimation of MRR-MDI-QKD in a Turbulent Channel

Consider the key rate formula of MDI-QKD [8]

(2) $$R\geq \mu_{a}\mu_{b}e^{-(\mu_{a}+\mu_{b})}Y_{11}^{Z}[1-h(e_{11}^{X})-Q_{\mu_{a}\mu_{b}}^{Z}f(E_{\mu_{a}\mu_{b}}^{Z})h(E_{\mu_{a}\mu_{b}}^{Z})]$$

Here, μ denotes the intensity of signal, and $Q_{\mu_{a}\mu_{b}}^{Z}$, $E_{\mu_{a}\mu_{b}}^{Z}$ are the gain and QBER, respectively, in the *Z* (signal) basis. H(x) represents the binary Shannon entropy. $Y_{11}^{Z,L}$ is the lower (upper) bound of a single photon yield. $e_{11}^{X,U}$ is the upper bound of the error rate of single photon states, which is estimated from the decoy state statistics in the X basis. Here, $Q_{\mu_{a}\mu_{b}}^{Z}$ and $E_{\mu_{a}\mu_{b}}^{Z}$ are simulated for rate estimation using known channel transmittance from Alice to Charlie, $\eta_{t_{a}}$ (Bob to Charlie, $\eta_{t_{b}}$), respectively, while in experiment they will be measured observables.

To obtain the channel transmittance, we first need to model the free-space channel. Different with a general free-space MDI-QKD, our MRR-MDI-QKD suffers from the double-pass channel, where the total geometric loss in the two passes can be described as [19,20]:

(3) $$\eta_{AC}=\frac{A_{Alice}A_{Charlie}}{\pi {\left(\frac{\theta_{A}}{2}\frac{\theta_{C}}{2}\right)}^{2}L_{AC}^{4}}\exp(-2\beta L_{AC}),\eta_{BC}=\frac{A_{Bob}A_{Charlie}}{\pi {\left(\frac{\theta_{B}}{2}\frac{\theta_{C}}{2}\right)}^{2}L_{BC}^{4}}\exp(-2\beta L_{BC})$$

Here, $\eta_{AC}$($\eta_{BC}$) represents the transmittance of Alice to Charlie (Bob to Charlie) affected by channel loss including atmospheric absorption and geometric spreading loss. $L_{AC}$($L_{BC}$) is the distance between Alice (Bob) and Charlie, β is the attenuation coefficient of the free-space link. $A_{Alice}$, $A_{Bob}$ and $A_{Charlie}$ denote the receiver apertures. $\theta_{A}$, $\theta_{B}$ and $\theta_{C}$ denote the angle divergences of the transmitter located in Alice, Bob and Charlie, respectively.

Due to the role of the MRR, this process involves the reflection of light. For this, let $\;I_{1}(I_{2})\;$ denote the forward-pass (backward-pass) channel coefficient. Taking the average of *N* samples, they can be formulated as [21]

(4) $$I_{1}=\sum_{i=1}^{N}\frac{U_{MMR}^{i}R_{ref}\left(\theta \right)}{\sum_{i=1}^{N}U_{in}^{i}circ(D_{tra})}$$

(5) $$I_{2}=\sum_{i=1}^{N}U_{out}^{i}circ(D_{tra})/\sum_{i=1}^{N}U_{MRR}^{i}R_{ref}(\theta )$$

Here, $\;U_{in}^{i},U_{MRR}^{i}\;$ are the amplitudes of the laser beams arriving at Alice (Bob)’s MRR, respectively. $\;U_{out}^{i}\;$is the amplitude of the laser beam received by Charlie, and $\;circ(D_{MRR})$ is the circular function related with the aperture diameter. Moreover, when the beam diameter  r(θ)  is small relative to the aperture, we set $circ(D_{MRR})\;$ as fixed. In addition, we assumed that  r(θ)  is the reflection ratio related with the incident angle with respect to the MRR. Therefore, the reflection effect of the MRR can be written as follows [22]:


(6) $$R_{ref}(\theta )=circ(D_{MRR})r(\theta )$$

With the above approximations, we now set $\;\eta_{a}\;$ as total reflection-induced transmission, which can be expressed by

(7) $$\eta_{a}=RI_{1}I_{2}$$

where the normalized reflection ratio of the MRR  R  is given by $\;R_{ref}(\theta )/R_{ref}(0)$.

Furthermore, the optical beam in such a double-pass configuration is jointly affected by the fluctuations of the refractive index (i.e., atmospheric turbulence) in the forward pass from the transceiver to the MRR and that in the backward pass from the MRR to the transceiver, which results in fluctuations in the channel transmittance [23]. To quantify the fluctuations, the Log-normal distribution is considered to characterize the type of weak turbulence conditions. Due to the assumption of uncorrelated fading between the forward and backward passes is not appropriate in the MRR-MDI-QKD configuration, we considered that the double-pass channel can be modeled by the distribution of the weighted product of two correlated Log-normal random variables. Based on the correlated Log-normal distribution with a correlation coefficient $\;\rho_{I}$, the probability distribution for the total turbulent-induced fluctuating transmission coefficient [24] is given as:

(8) $$p(\eta_{a})=\frac{1}{2\sqrt{2\pi }\eta_{a}\sigma_{xt}}\exp\left(-\frac{{\left(\ln\eta_{a}-\ln R-2\mu_{xt}\right)}^{2}}{8\sigma_{xt}^{2}}\right)$$

Here, the corresponding $\;\mu_{xt}\;$ and $\;\sigma_{xt}\;$ are given as:

(9) $$\begin{array}{l} \mu_{xt}=\frac{1}{2}\ln\mu_{1}\mu_{2}-\frac{1}{4}\ln((\frac{\sigma_{1}^{2}}{\mu_{1}^{2}}+1)(\frac{\sigma_{2}^{2}}{\mu_{2}^{2}}+1)),\\ \sigma_{xt}=\sqrt{\frac{1}{4}\ln(\frac{\sigma_{1}^{2}}{\mu_{1}^{2}}+1)+\frac{1}{4}\ln(\frac{\sigma_{2}^{2}}{\mu_{2}^{2}}+1)+\frac{1}{2}\rho_{x}\sqrt{\ln(\frac{\sigma_{1}^{2}}{\mu_{1}^{2}}+1)\ln(\frac{\sigma_{2}^{2}}{\mu_{2}^{2}}+1)}}\\ \rho_{x}=1/\sqrt{\ln(\sigma_{1}^{2}/\mu_{1}^{2}+1)\ln(\sigma_{2}^{2}/\mu_{2}^{2}+1)}\ln(\rho_{I}\sigma_{1}\sigma_{2}/(\mu_{1}\mu_{2})+1) \end{array}$$

Therefore, the total channel transmittance can be respectively defined as:

(10) $$\eta_{t_{a}}=\eta_{a}\eta_{AC}$$

To summarize, the secure key rate of our scheme can be obtained as follows:

(11) $$R(\eta )=\int_{0}^{1}\int_{0}^{1}p\left(\eta_{t_{a}}\right)p\left(\eta_{t_{b}}\right)R(\eta_{t_{a}},\eta_{t_{b}})d\eta_{t_{a}}d\eta_{t_{b}}$$

## 4. Results

In this work, we focus on the symmetric case where the two channel transmissions from Alice to Charlie and from Bob to Charlie are equal. Since we only concentrate on the high-loss region, the geometric loss is ranged from 25 dB~55 dB for convenience. Here, we first compare the key generation rate and QBER using the correlated Log-normal distribution (Correlated-LD) to those using the Log-normal distribution (LD) presented in [24], which are both applicable to the weak turbulence channel. Below for simplicity, we fixed the reflection effect in Equation (6) at 1, which corresponds to the perfect alignment between Alice and Bob. The forward-pass and the backward-pass channel coefficients $I_{1}$ and $I_{2}$ are also set as 1, respectively. In addition, the signal and decoy state intensity in MDI-QKD setups are fixed at 0.3 and 0.05, respectively. Other numerical parameters are chosen from [25], which are listed in Table 1.

**Figure 3:**
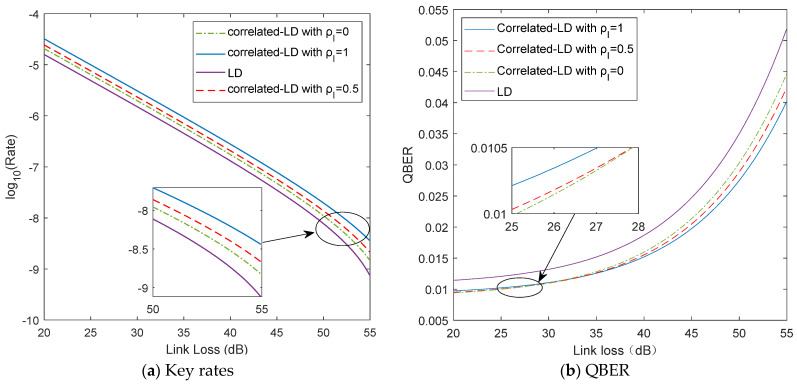
Key rates and QBER versus channel loss in the two different distributions. For a comparison, the fading correlation coefficient $\;\rho_{I}\;$ ranges from 0 to 1, and the turbulence intensity in the forward-pass $\;\sigma_{1}\;$ (the backward-pass $\;\sigma_{2}$) are fixed at 0.3.

The curves of and the key rate and QBER in the asymptotic case for the two different distributions mentioned above are shown in Figure 3a and Figure 3b, respectively. In Figure 3a, we observe that, in the case with $\;\rho_{I}=1$ (i.e., a strict correlation between uplink and downlink), the secret key generation rate in the correlated Log-normal distribution is closer to those in the Log-normal distribution. Furthermore, as shown in Figure 3b, the estimated parameter of QBER clearly shows that the correlated Log-normal distributions are more tightly distributed than the Log-normal distribution, which leads to a higher key rate. The remarkable behavior is that, by scanning through the decoy-state intensities and probabilities, the key rate, in fact, can be further optimized [8,9]. Hence, the correlated Log-normal distributions can give more practical secret key generation estimation.

**Figure 4:**
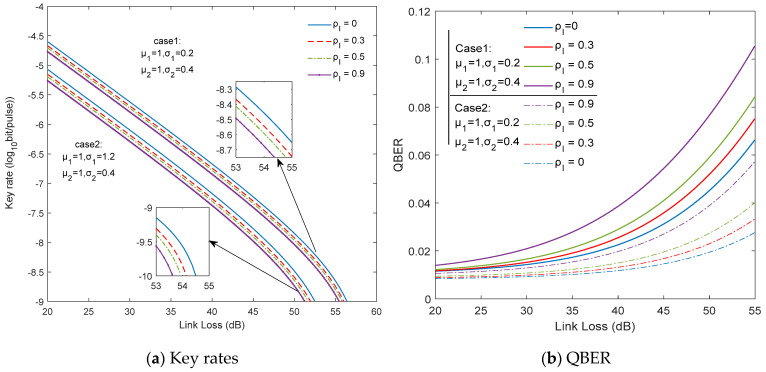
Key rates and QBER versus channel loss in the two different weak turbulence cases. The fading correlation coefficient $\;\rho_{I}\;$ ranges from 0 to 0.9. The two different values set for the turbulence intensity $\;\sigma_{1}\;$ and $\;\sigma_{2}\;$ as {0.4, 0.2} and {0.4, 1.2} were presented in our simulation. When the double-pass channel parameters $\mu_{1}$, $\sigma_{1}$, $\mu_{2}$, $\sigma_{2}$ are fixed, the performance is impaired by the fading correlation coefficient $\;\rho_{I}$ and deteriorating with increasing $\;\rho_{I}$. When the fading correlation coefficient $\;\rho_{I}$ is fixed, the key rates of our scheme increase with the decrease of the turbulence intensity $\;\sigma_{2}\;$.

We then numerically simulate the performance of our protocol using the correlated Log-normal distributions with different turbulence intensities. The secret key generation rate and QBER in two different weak turbulence cases are show in Figure 4a,b, respectively. For higher channel loss the averaged key rate significantly decreases and the QBER increases. However, we found that there was a slight drop of the key rates, as shown in Figure 4a, when the fading correlation coefficient $\;\rho_{I}\;$ increases from 0 to 0.9. Hence, under long-distance propagation distance, the main factor that causes attenuation is still the geometric spreading loss. On the other hand, as shown in Figure 4b, it is a remarkable fact that at a higher channel loss, the QBER in two different turbulence intensity cases grows at different rates, thereby varying degrees of impact on the key rate. This is because the turbulence-induced fluctuating transmittance $\;\eta_{a}\;$ is related with $\;E_{uu}(\eta_{t})\;$ and the turbulence intensity  σ. Hence the effect of the fading correlation coefficient $\rho_{I}$ on QBER is weak when the channel loss tends to zero and becomes larger with an increase of the product term $\eta_{l}$, which is in agreement with the previous discussion.

## 5. Conclusions

In this paper, a modified MDI-QKD scheme with modulating retro-reflectors is proposed not only to inherit the merit of the structure in which the experimental system is automatically stabilized in spectrum and polarization modes, but also to bring advantages of the MRR that simplify the pointing requirements due to its wide reflective field of view. Here, the double-pass correlated turbulent channel model is used to investigate the complex and unstable channel characteristics caused by the atmospheric turbulence. The simulation results clearly show that the correlated Log-normal distribution is more appropriate to characterize the correlated fading in the MRR-MDI-QKD, which could be used as a modeling method when estimating secret key rate in such free-space QKD configuration. Moreover, inspired by the idea of the Plug-and-Play MDI-QKD, the security analysis of MRR-MDI-QKD is analyzed under some classical attacks, which ensures that the MRR-MDI-QKD can be implemented with only ordinary optical elements in the experiment. Our work provides a meaningful step towards an MDI-QKD based on the moving platforms to join a dynamic quantum network with untrusted relays.

## Figures and Tables

**Table 1:** Lists of Necessary Parameters.

Symbol	Name	Value
$\eta_{d}$	detection efficiency	50%
$e_{0}$	error probability of dark counts	0.5
$e_{d}$	error probability of optical misalignment	0.015
$\;f_{e}$	error-correction efficiency	1.16
$Y_{0}$	background rate	3 × 10^−6^

## Data Availability

Data is contained within the article.

## Appendix A. Security Analysis

The MRR-MDI-QKD links share the same characteristics of general plug-and-play QKD links, and as a result also were faced with similar security issues. In this section, we give priority to consider the security implications of untrusted light sources caused by the structure. A complete proof of the unconditional security of MDI-QKD with an untrusted source is derived in [2], which considered various real-life imperfections in its implementation. Moreover, a general formalism is proposed to prove the security of MDI-QKD with leaky sources to relax this unrealistic assumption [26]. Specifically, there is no information leakage from the transmitters to the senders, which shows that MDI-QKD is feasible within a reasonable time frame of signal transmission given that the sources are sufficiently isolated. Reference [27] demonstrated a proof-of-principle experiment over an asymmetric 36 km fiber channel to improve the feasibility of plug-and-play MDI-QKD and discuss the methods to improve security by increasing active monitoring.

In practice, Bob limits his interrogation power and Alice adjusts the double pass loss of the MRR array to change the mean number of photons that are retro-reflected to implement the decoy state method, which is usually used to defend against photon number splitting attacks. The intercept-resend attacks can be defeated with active countermeasures, where Alice also uses the photo detection capability to monitor the incoming power of all four MRRs. The Trojan horse attack, which is the main threat in plug and play systems, can be effectively defended by increasing active monitoring to detect the incoming optical power. Though our modified plug-and-play MDI-QKD scheme with MRR is vulnerable to the source attacks also facing in plug-and-play QKD schemes, the unique characteristics of the MRR devices allow them to act as both power and angle of arrival detectors without adding extra components to the system.
